# A case of absent right and persistent left superior vena cava

**DOI:** 10.1186/1476-7120-4-6

**Published:** 2006-01-26

**Authors:** Attila Pálinkás, Edit Nagy, Tamás Forster, Zita Morvai, Endre Nagy, Albert Varga

**Affiliations:** 1 Department of Internal Medicine, Elisabeth Hospital, Hódmezõvásárhely, Hungary; 2 2nd Department of Internal Medicine and Cardiology Center, University of Sciences, Szeged, Hungary

## Abstract

**Background and purpose:**

Our case report deals with the importance of detailed echocardiographic examination for differential diagnosis of coronary sinus dilation and development of abnormalities of great thoracic veins.

**Case presentation:**

A 49-year-old man underwent transthoracic echocardiography for atypical chest pain. A dilated coronary sinus was found and venous contrast echocardiography raised the suspicion of absent right and persistent left superior vena cava. Transesophageal echocardiography showed absence of right superior vena cava. The echocardiographic findings were confirmed by upper venous digital subtraction cavography.

**Conclusion:**

combination of agenesia of right SVC and isolated persistent left SVC in adult patients is a very rare abnormality. Both clinicians and sonographers should be alerted to the possible presence of this combined venous anomaly. Transthoracic echocardiograpy – including agitated saline infusion to the antecubital vein – is an important diagnostic tool for accurate diagnosis of this congenital thoracic venous malformation.

## Background

The presence of left superior vena cava (SVC) occurs in approximately 0.3% to 0.5% of the general population [1,2]. In most of patients with left SVC right SVC is present. However very rarely right SVC may be absent and in these cases a persistent left SVC drains the venous blood of both upper extremities and the head into the heart. We present one case of isolated persistent left SVC with absence of right SVC. Our case report deals with the importance of detailed echocardiographic examination for differential diagnosis of coronary sinus dilation and development abnormalities of great thoracic veins.

## Case presentation

**Figure 1:**
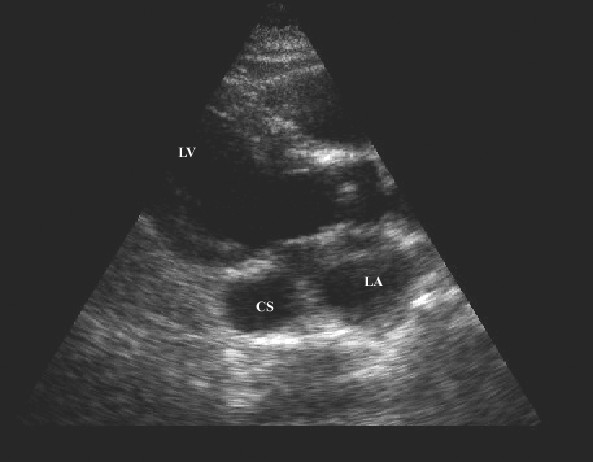
Parasternal long axis two-dimensional echocardiographic view showing dilated coronary sinus. (LA = left atrium, LV = left ventricle, CS = coronary sinus).

**Figure 2:**
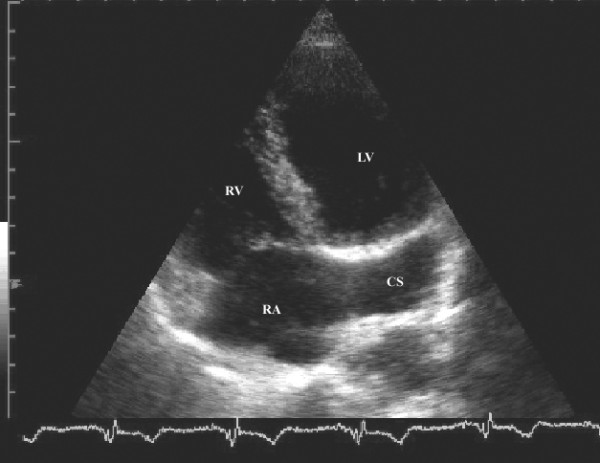
Modified apical 4 chamber two-dimensional echocardiographic view showing dilated coronary sinus with 2.8 cm in diameter. (LA = left atrium, LV = left ventricle, CS = coronary sinus)

**Figure 3:**
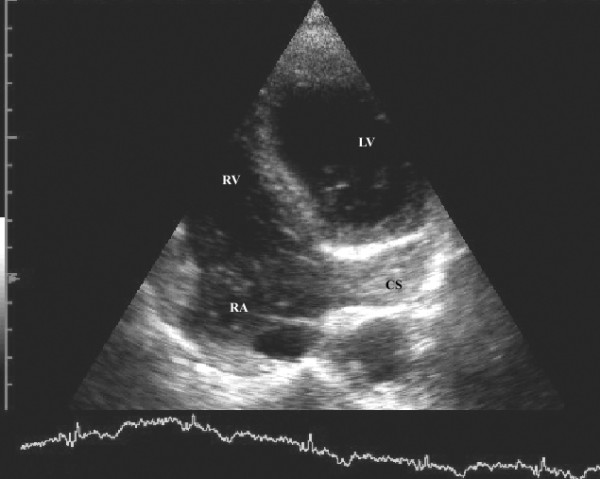
Injection of agitated saline into both left and right antecubital vein results in filling of coronary sinus first followed by the filling of the right atrium (LA = left atrium, LV = left ventricle, CS = coronary sinus).

**Figure 4:**
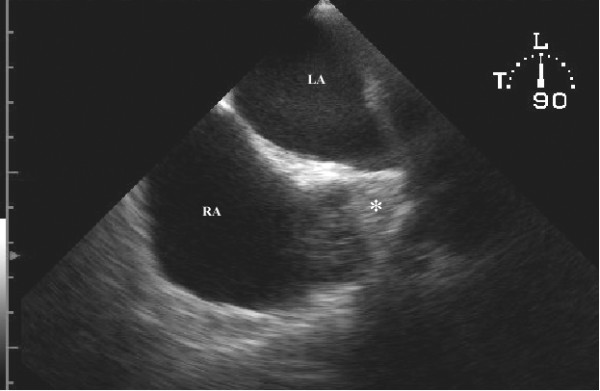
Transesophageal echocardiography in right atrial long axis two-dimensional echocardiographic view demonstrates absence of right vena cava superior (black star) (LA = left atrium, RA= right atrium).

**Figure 5:**
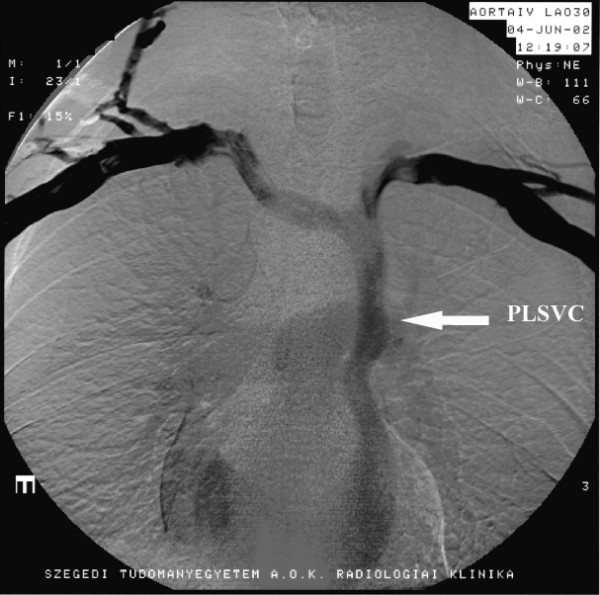
Upper venous digital subtraction cavography which indicates absence of the right superior vena cava and a persistent left superior vena cava (PLSVC) in the left lateral part of the thorax (white arrow).

A 49-year-old man was hospitalized due to pleuropneumonia. Transthoracic echocardiography in parasternal and modified apical view showed a markedly dilated coronary sinus (Figures 1 and 2). There was no evidence of valvular heart disease and diameters of cardiac chambers were within normal limits. Systolic and diastolic function of the left ventricle and estimated pulmonary systolic pressure were in normal range. All pulmonary veins drained into the left atrium. In order to reveal the cause of coronary sinus dilation an agitated saline injection was given into the left antecubital vein. The contrast entered first into the coronary sinus and subsequently appeared in the right atrium. Similarly, when the saline injection was given into the right antecubital vein, the contrast agent appeared first in the dilated coronary sinus and thereafter entered into the right atrium (Figure 3, additional file 1). Transesophageal echocardiography showed absence of right SVC, presence of left SVC at lateral border of left atrium and no evidence of any other structural abnormalities of the heart and great thoracic vessels (Figure 4additional file 2). An upper venous digital subtraction cavography confirmed the absence of the right SVC and the presence of persistent left SVC (Figure 5). Surface electrocardiogram and laboratory exams were within normal limits. Abdominal sonography verified normal position and structure of visceral organs.

## Discussion

Left SVC is a relatively rare congenital venous development anomaly occurring in approximately 0.3% to 0.5% of the general population [1,2]. It incidence is ten fold higher in patients with congenital heart malformation [2,3]. During normal cardiac development the left-sided anterior venous cardinal system disappears, leaving only the coronary sinus and a remnant known as the ligament of Marshall. Failure of closure of the left anterior cardinal vein results in persistent left SVC. In vast majority of cases the persistent left SVC drains into the right atrium via an enlarged coronary sinus. On rare occasions, the persistent left SVC enters directly into the left atrium, resulting in partial anomalous systemic venous return [4]. Besides being associated with other congenital heart malformations, the most relevant clinical implication of left SVC is the association with disturbances of cardiac impulse formation and conduction [5]. Persistent left SVC is generally associated with right SVC and very rarely with absent right SVC. In these rare instances both right and left brachiocephalic vein drain into the persistent left SVC at the leftinternal side of the thorax. Bartram et al. reviewed 121 patients with persistent left SVC and absent right SVC [6]. About half of these cases had no other congenital malformations. Most common congenital defects associated with combined great venous malformation were various types of atrial septal defects, endocardial cushion defects and tetralogy of Fallot. Other reported co-existent congenital anomalies were coarctation of aorta, ventricular septal defect, double aortic arch, transposition of great arteries, dextrocardia and asplenia. In our case an echocardiographically detected dilated coronary sinus raised the suspicion of con genital heart malformation. Simple agitated saline bolus injections into the antecubitalveins can add an important clue for differential diagnosis of coronary sinus dilation. When scanning healthy individuals in modified four-chamber view the saline contrast administered thorough both upper extremity vein first appear in the right atrium. The contrast given into the left antecubital vein will first appear in the dilated coronary sinus then in the right atrium, if there is a persistent left SVC that drains to the coronary sinus. In rare cases of right SVC agenesia and persistent left SVC the contrast enters first into the markedly dilated coronary sinus from both brachial vein, as in our case. Transesophageal echocardiographic examination can add further information on possible co-existent congenital heart defects causing coronary sinus dilation and verify agenesia of right SCV [7]. Venous angiography is a widely available and low cost imaging method for definite confirmation of the presented combined venous malformation. Other alternative imaging methods include computed tomography and magnetic resonance imaging scan [8,9]. The hemodynamics of patients with agenesia of right SVC and isolated persistent left SVC connected to the right atrium is the same as that of health y individuals, as a result, patients are mostly asymptomatic. However it may cause difficulties and serious complications in central venous catheterization, intracardiac electrode placement or during cardiopulmonary bypass [10]. Detailed and accurate echocardiographic studies may identify this rare combined congenital defect, hence preventing future complications during invasive procedures.

## Conclusion

Combination of agenesia of right SVC and isolated persistent left SVC in adult patients is a very rare anomaly. Both clinicians and sonographers should be alerted to the possible presence of this combined vnous anomaly. Transthoracic echocardiograpy – including agitated saline infusion to both antecubital vein – is an important diagnostic tool for accurate diagnosis of this rare congenital thoracic venous malformation.

## Competing interests

Authors have read and approved submission of the manuscript, and no conflict of interest exists for any of the authors

## Authors' contributions

AP carried out the contrast echo examination and wrote the paper

EdN did the baseline echo and followed the patient

TF supervised the TEE

ZM and EnN performed the digital subtraction cavography

AV carried out the TEE and wrote the paper

## Supplementary Material

Additional File 1 Echo contrast in the right atrium via sinus coronaries. Click here for file

Additional File 2 Transesophageal clip demonstrating the absence of right vena cava superior. Click here for file
